# IK acts as an immunoregulator of inflammatory arthritis by suppressing T_H_17 cell differentiation and macrophage activation

**DOI:** 10.1038/srep40280

**Published:** 2017-01-10

**Authors:** Hye-Lim Park, Sang-Myeong Lee, Jun-Ki Min, Su-Jin Moon, Inki Kim, Kyung-Won Kang, Sooho Park, SeulGi Choi, Ha-Na Jung, Dong-Hee Lee, Jae-Hwan Nam

**Affiliations:** 1 Department of Biotechnology, The Catholic University of Korea, Bucheon, 420-743, Korea; 2 Division of Biotechnology, Advanced Institute of Environment and Bioscience, College of Environmental and Bioresource Sciences, Chonbuk National University, Iksan, 570-752, Korea; 3 Division of Rheumatology, Department of Internal Medicine, College of Medicine, The Catholic University of Korea, Seoul,137-040, Korea; 4 Department of Medicine, College of Medicine, University of Ulsan, Seoul 138-736, Korea; 5 Biomaterials Research Center, Cellinbio, Suwon, 443-734, Korea

## Abstract

Pathogenic T helper cells (T_H_) and macrophages have been implicated in the development of rheumatoid arthritis (RA), which can lead to severe synovial inflammation and bone destruction. A range of therapies have been widely used for RA, including specific monoclonal antibodies and chemical inhibitors against inflammatory cytokines produced by these cells. However, these have not been sufficient to meet the medical need. Here, we show that in transgenic mice expressing truncated IK (tIK) cytokine, inflammatory arthritis symptoms were ameliorated as the result of suppression of the differentiation of T_H_1 and T_H_17 cells and of macrophage activation. During inflammatory responses, tIK cytokine systemically regulated macrophage functions and T_H_17 cell differentiation through inactivation of the MAPK and NF-κB pathways. Interestingly, the level of tIK cytokine was higher in synovial fluid of RA patients compared with that in osteoarthritis (OA) patients. Our observations suggest that tIK cytokine can counterbalance the induction of inflammatory cells related to RA and thus could be a new therapeutic agent for the treatment of RA.

Rheumatoid arthritis (RA) is an acute-on-chronic, systemic autoimmune disease that affects about 1% of the population^1^. The pathology of RA is characterized by infiltration of inflammatory cells into the pannus and the synovial fluid, and by ensuing tissue destruction^1^,^2^,^3^. Moreover, it is known that an imbalance between pro- and anti-inflammatory cytokines can lead to the induction of a chronic inflammatory process and subsequent joint destruction. In particular, CD4^+^ T helper cells that express interferon (IFN)-γ and interleukin (IL)-17 (T_H_1 and T_H_17 cells, respectively) and macrophages, which infiltrate the synovium, are considered to be the main drivers in the pathogenesis of RA^4^,^5^,^6^,^7^. In addition, self-antigen presentation through aberrant major histocompatibility complex (MHC) class II-expressing B cells produces autoantibodies, leading to the development of more erosive RA^2^,^8^. Recently, based on increased understanding of the immune cells and inflammatory cytokines involved in pathogenesis of RA, various therapies have been applied to RA treatment, including specific monoclonal antibodies against RA-related cytokines and chemical inhibitors of RA-related signal pathways^3^,^5^. However, an inhibitory cytokine that could maintain homeostasis to ameliorate inflammatory autoimmune RA has not yet been identified.

Inhibitor of K562 (IK) cytokine was reported as a novel inhibitor of both IFN-γ-induced and constitutive expression of MHC class II molecules on B cells^9^. The IK cytokine harvested from the supernatant of K562 was truncated to a 19-kd protein (truncated IK, tIK cytokine), which was translated from the methionine 316 residue of the full-length IK cytokine without the nuclear localization sequence^10^. This tIK cytokine still functioned effectively in downregulation of MHC class II expression, similarly to the full-length IK cytokine^11^,^12^. Moreover, it protected against systemic lupus erythematosus pathogenesis by reducing MHC class II expression and anti-DNA antibodies^11^. Recently, we reported that coxsackievirus B3 (CVB3), which can induce systemic activation of most immune cells, producing a cytokine storm, transiently induced IK cytokine expression and was also able to downregulate expression of MHC class II on B cells by increasing cAMP^12^. Based on these reports, it can be speculated that tIK cytokine may regulate excessive activation of immune cells. However, the immunological mechanism of tIK cytokine and its effects in other autoimmune diseases such as RA have not yet been determined.

Here, we investigate the functional effect of tIK cytokine in inflammatory processes and inflammatory arthritis. We show that tIK cytokine suppressed activation of macrophages and the differentiation of T_H_1 and T_H_17 cells in a mouse model of inflammatory arthritis. Moreover, we found that tIK cytokine inhibited LPS-triggered inflammation. These findings indicate that tIK cytokine can function, at least partially, to prevent the induction of inflammatory cytokines including IL-17, and therefore it could possibly ameliorate the progression of joint inflammation and damage in RA.

## Results

### tIK cytokine alleviates inflammatory arthritis in a mouse model of inflammatory arthritis

**Figure 1:**
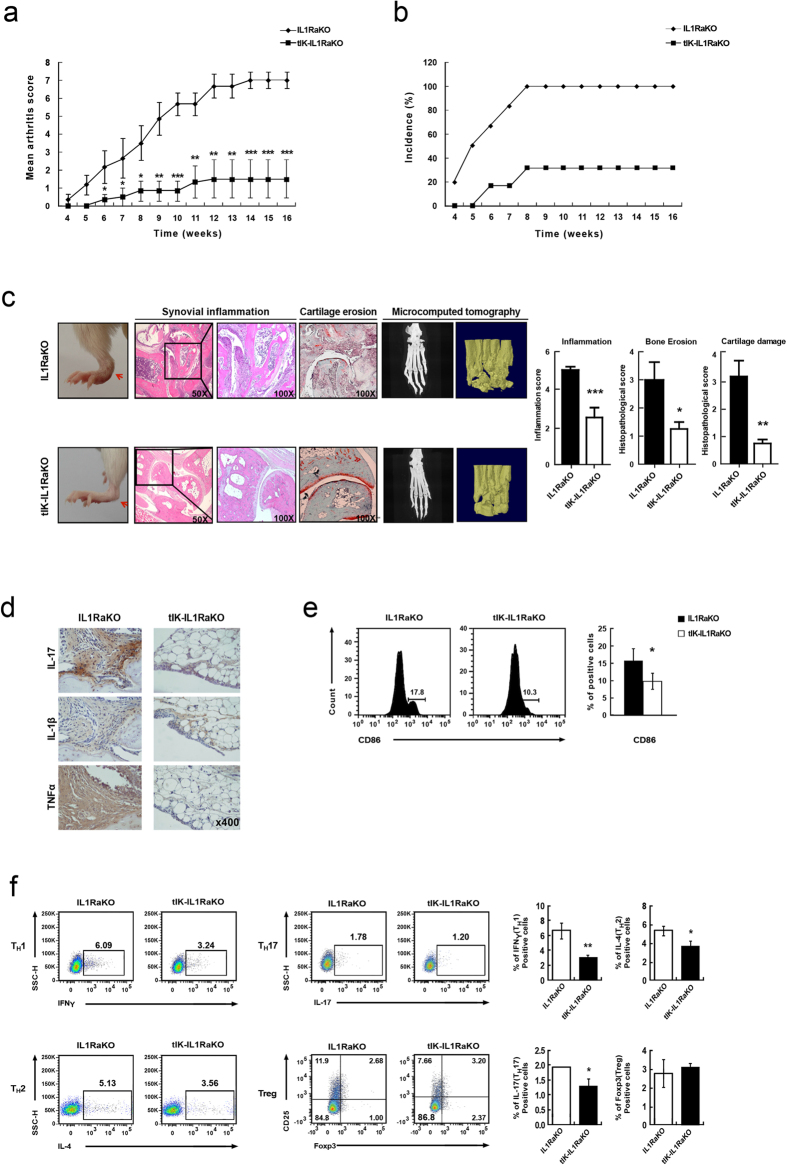
tIK cytokine ameliorates the symptoms in a mouse model of inflammatory arthritis. (**a**,**b**) Severity score and incidence of arthritis in IL1RaKO and tIK-IL1RaKO mice (n = 10, mean ± SD). (**c**) The paw and hematoxylin and eosin- (synovial inflammation, original magnification, x50 and x100), safranin O- (cartilage erosion, original magnification, x100), stained representative joint sections in IL1RaKO and tIK-IL1RaKO mice (n = 6) at 16 weeks old. The red arrows point to the arthritic joint. The graphs on the right represent the histological scores for inflammation and cartilage erosion (n = 6, mean ± SD). Microcomputed tomography of the joints in IL1RaKO and tIK-IL1RaKO mice (n = 3) at 16 weeks old. (**d**) Inflammatory cytokines (original magnification, x400)-stained representative joint sections in IL1RaKO and tIK-IL1RaKO mice (n = 3) at 16 weeks old. (**e,f**) Flow cytometric analyses of expression of CD86 in CD11b^+^ F4/80^+^ macrophages (**e**) and IFN-γ, IL-4, and IL-17 in CD4^+^ T cells (**f**) from splenocytes in IL1RaKO and tIK-IL1RaKO mice. The graphs indicate frequencies of gated cells (n = 4, mean ± SD). Statistical significance was determined by the two-tailed unpaired Student t-test. *P < 0.05, **P < 0.01, ***P < 0.001.

**Figure 2:**
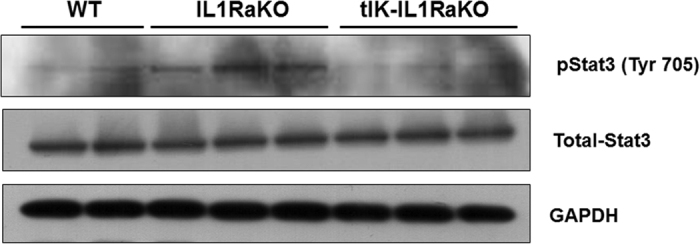
T_H_17 related molecule was inhibited in tIK-IL1RaKO mice. Western blot analysis of STAT3 and GAPDH in splenic T cells from IL1RaKO and tIK-IL1RaKO mice. The original blots were cropped by Adobe photoshop. Full-length blots are presented in Supplementary Figure 8.

To investigate the potential of tIK cytokine in RA, we generated a crossbred mouse by breeding a tIK-expressing transgenic (termed tIK-Tg) mouse^12^ and an IL-1 receptor antagonist knockout (termed IL1RaKO) mouse on the BALB/c background^13^,^14^,^15^. This crossbred mouse was designated tIK-IL1RaKO (Fig. S1). IL-1RaKO mice on the BALB/c background develop polyarthritis spontaneously^13^. In these mice, excess IL-1 signaling leads to T cell-mediated autoinflammation and the development of inflammatory arthritis, suggesting that this animal model closely resembles human RA. Therefore, to evaluate the effects of tIK cytokine in inflammatory arthritis, we assessed joint swelling and the incidence of arthritis by visual examination of the paws of both tIK-IL1RaKO and IL1RaKO mice up to 16 weeks of age. Interestingly, over the entire period, tIK-IL1RaKO mice had significantly lower arthritis scores than IL1RaKO mice (Fig. 1a). In addition, at 16 weeks, arthritis had developed in only 30% (3 of 10) of the tIK-IL1RaKO mice compared with 100% (10 of 10) of the IL1RaKO mice (Fig. 1b). We also observed very mild paw swelling with some inflammatory cell infiltration and less severe cartilage erosion in most of the joints of tIK-IL1RaKO mice compared with the joints of IL1RaKO mice (Fig. 1c). Consistent with these findings, tIK-IL1RaKO mice showed only mild bone destruction and lower expression of inflammatory cytokines in their joints (Fig. 1c,d). The tIK-IL1RaKO mice expressed high levels of tIK cytokine mRNA and significantly reduced levels of inflammatory cytokines in the spleen, joints, and serum (Fig. S2a–c). Furthermore, we found that tIK-IL1RaKO mice showed lower percentages of IFN-γ-expressing, IL-4-expressing, and lower CD86^+^ expression on macrophages and IL-17-expressing CD4^+^ T helper cells (T_H_1, T_H_2, and T_H_17 cells, respectively) compared with the IL1RaKO mice (Fig. 1e,f). In addition, although the levels of effector CD4^+^ T cells and B220^+^ IgD^+^ B cells in the spleens of tIK-IL1RaKO mice were slightly reduced (Fig. S3a,b), the development of T cells and activation of dendritic cells did not differ between the mouse groups (Fig. S3c,d). It is known that signal transducer and activator of transcription 3 (STAT3) plays critical roles in the development and proliferation of T_H_17 cells^16^. We found that the expression of STAT3 in splenic T cells of tIK-IL1RaKO mice was reduced compared with that in IL1RaKO mice (Fig. 2). These findings clearly indicate that tIK cytokine can protect against the progress of inflammatory arthritis in a mouse model, suppressing inflammation by interrupting the development of activated pathogenic immune cells, especially T_H_17 and T_H_1 cells and macrophages.

### tIK cytokine attenuates inflammation-induced RA risk factors

**Figure 3:**
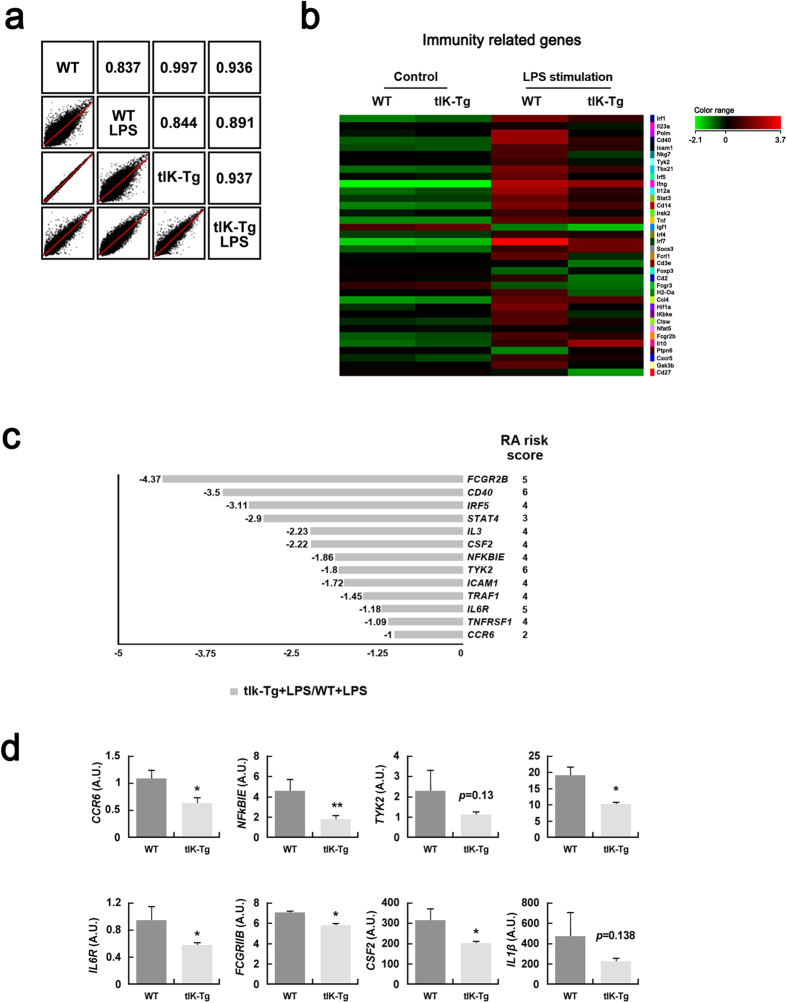
tIK cytokine attenuates LPS-induced inflammatory responses in immune cells. (**a**) Scatter plots showing pairwise comparison between splenocytes of WT and tIK-Tg mice under LPS stimulation. (**b**) Microarray analysis of splenocytes in the presence or absence of LPS treatment. The heat map shows a selection of 36 up- and downregulated genes (mean fold change in two independent experiments). (**c**) Comparison of RA risk factor genes in LPS-stimulated splenocytes of WT and tIK-Tg mice. The bar graphs show the fold changes in expression of genes for risk factors selected by their RA risk score (LPS-stimulated tIK-Tg/LPS-stimulated WT mice). (**d**) Analysis of representative expression of mRNA for immunity- and defense-related genes including RA risk factors in LPS-stimulated splenocytes from WT and tIK-Tg mice. Results are representative of three experiments. A.U. = arbitrary units. Statistical significance was determined by the two-tailed unpaired Student t-test. *P < 0.05, **P < 0.01.

To examine the immune regulatory function of tIK cytokine under inflammatory conditions, we performed a microarray analysis using samples of total RNA from LPS O111:B4-treated splenocytes of wild-type (WT) and tIK-Tg mice. We found that the gene expression profiles of both strains (WT versus tIK-Tg) in the steady state (no treatment with LPS) were very similar (0.997; Fig. 3a and Fig. S4a). However, those in LPS-treated splenocytes of tIK-Tg and WT mice were significantly different (0.891, WT-LPS versus tIK-Tg-LPS; Fig. 3a). This suggests that tIK cytokine may only function under stressed/stimulated conditions, but not under normal conditions. In fact, WT mice showed considerable changes in their gene expression profile after LPS treatment (0.837, WT versus WT-LPS; Fig. 3a), whereas tIK-Tg mice showed some resistance to LPS-induced changes (0.937, tIK-Tg versus tIK-Tg-LPS; Fig. 3a). Specifically, some immunity-related genes were significantly resistant to LPS stimulation in the tIK-Tg mice (Fig. 3b). Among these, expression of RA risk-related genes^17^ was reduced in the tIK-Tg mice (Fig. 3c), and this reduction was verified by real-time PCR (Fig. 3d). These data indicate that tIK cytokine confers systemic resistance to an inflammatory stimulus and the induction of RA-related genes. Moreover, we did verify the expression of inflammatory cytokines in splenocytes of IL1RaKO and tIK-IL1RaKO mice stimulated for 16 h with LPS (O111:B4, 100 ng/ml). After LPS stimulation, the splenocytes of tIK-IL1RaKO mice showed lower expression of TNFα and IL1β than those of IL1RaKO mice. Therefore, RA risk genes play an important role in our experimental animal model of arthritis (Fig. S4d).

### tIK cytokine regulates macrophage signaling and functional effects

**Figure 4:**
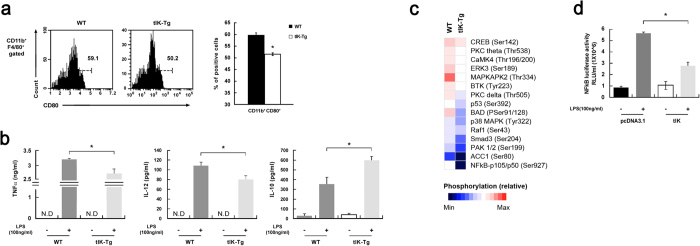
tIK cytokine inhibits macrophage signaling and functional effects. (**a**) Expression of splenic macrophage surface marker (CD11b) and costimulatory factor (CD80) in 2.5 mg/kg LPS-challenged WT and tIK-Tg mice. The graph represents the frequencies of gated cells among the total splenocytes (n = 3, mean ± SD). (**b**) Cytokine production by BMDMs from WT and tIK-Tg mice stimulated with LPS (100 ng/ml) for 24 h. Cell culture supernatants were analyzed for TNF-α, IL-12, and IL-10 by ELISA (n = 3, mean ± SD). (**c**) Heat map of Phospho Explorer array analysis using LPS-stimulated BMDMs from WT and tIK-Tg mice. The color index indicates the ratio of phosphorylation intensity. Results are representative of three independent experiments. (**d**) NF-κB promoter activity of tIK gene-transfected Raw 264.7 macrophage cells treated with LPS (100 ng/ml) for 24 h (n = 3, mean ± SD). Statistical significance was determined by the two-tailed unpaired Student t-test. *P < 0.05.

To corroborate the above findings, we injected 2.5 mg/kg doses of LPS into WT and tIK-Tg mice. The tIK-Tg group showed lower mortality (57.2% of WT versus 28.6% of tIK-Tg), and relatively lower macrophage activation at 24 h after exposure to LPS (Fig. 4a). Next, we treated bone-marrow-derived macrophages (BMDMs) from WT and tIK-Tg mice with LPS. BMDMs from tIK-Tg mice at 24 h after LPS treatment showed reduced production of inflammatory cytokines (Figs 4b and S4b), but induction of the anti-inflammatory cytokine IL-10 (Fig. 4b). Raw 264.7 macrophage cells transfected with the tIK cytokine gene also showed reduced expression of inflammatory cytokine genes (Fig. S4c). Interestingly, a protein kinase array also showed a reduction of the phosphorylation of some inflammation-related factors, including MAPK and NF-κB intermediates, in LPS-stimulated BMDMs from tIK-Tg mice (Fig. 4c). In addition, the promoter activity for NF-κB was significantly lower in tIK-transfected Raw 264.7 cells than in pcDNA3.1-transfected cells at 24 h after stimulation with LPS (Fig. 4d). These results indicate that tIK cytokine plays at least a partial role in acute protection against inflammation caused by macrophage activation.

### tIK cytokine affects differentiation of pathogenic T_H_17 cells in the steady state

**Figure 5:**
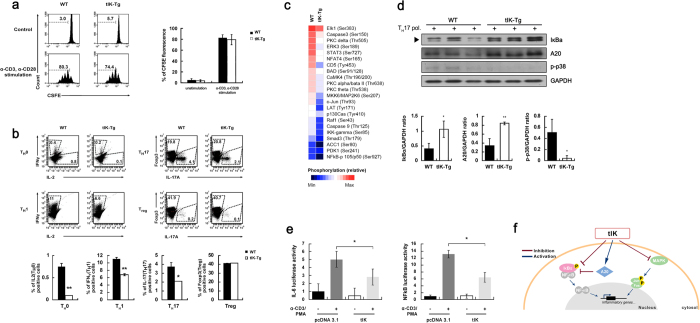
tIK cytokine suppresses pathogenic T_H_17 cell differentiation. (**a**) Proliferation of CD4^+^ T cells from WT and tIK-Tg splenocytes stimulated with antibodies to CD3 and CD28 (n = 4, mean ± SD). (**b**) Differentiation of CD4^+^ T cells from WT and tIK-Tg mice stimulated with antibodies to CD3 and CD28 under T_H_0-, T_H_1-, or T_H_17-polarizing conditions. Intracellular expression of IL-2, IFN-γ, or IL-17 was detected by flow cytometry. All graphs represent the frequencies of gated cells among CD4^+^ splenic T cells (n = 4, mean ± SD). (**c**) Heat map of Phospho Explorer array analysis using T_H_17-polarized splenic T cells from WT and tIK-Tg mice. The color index indicates the ratio of phosphorylation intensity. (**d**) Western blot analysis of IκBα, p-p38, A20, and GAPDH in CD4^+^ splenic T cells from WT and tIK-Tg mice stimulated under T_H_17-polarizing conditions. The bar graphs below show the ratios of the band intensities normalized to that of GAPDH. Results are representative of three independent experiments. The original blots were cropped by Adobe photoshop. Full-length blots are presented in Supplementary Figure 9. (**e**) Promoter activities of NF-κB and IL-6 in tIK gene-transfected Jurkat T cells stimulated with PMA (10 ng/ml) and anti-CD3 (1 μg/ml) for 18 h. Statistical significance was determined by the two-tailed unpaired Student t-test. *P < 0.05, **P < 0.01.

In tIK-Tg mice, we observed that the mRNA of tIK cytokine was more highly expressed in lymphoid organs such as the lymph nodes, spleen, and thymus than in non-lymphoid organs (Fig. S5a). This means that we can use tIK-Tg mice to test the function and mechanism of endogenous tIK cytokine in adaptive immune cells. In the steady-state model, development and activation of T cells, macrophages, and dendritic cells, and proliferation of B cells from the spleen did not differ substantially between the WT and tIK-Tg mice (Figs S5 and 6). However, the total number of splenic B cells and the development of B cells were slightly impaired in tIK-Tg mice compared with WT mice (Fig. S5d). Furthermore, despite similar levels of proliferating T cell populations (Fig. 5a), the differentiation of T_H_0, T_H_1, and T_H_17 cells was suppressed in tIK-Tg mice (Fig. 5b). These results indicate that tIK cytokine significantly impairs T cell differentiation. These suppressive effects of tIK cytokine on immune cells may suggest an increase in the risk of pathophysiology because of impaired immunity. However, the major organs of tIK-Tg mice showed similar histology at 4 weeks of age to those of the WT mice. Moreover, 1-year-old tIK-Tg mice also showed normal histology (Fig. S7). Thus, tIK cytokine functions only under stressed/stimulated conditions (Fig. 3a), meaning that it may specifically act as a counterbalance to induction of inflammatory conditions.

To explore the mechanism by which tIK inhibits T_H_17 cell differentiation, we stimulated splenic CD4^+^ T cells from WT and tIK-Tg mice under T_H_17 cell-differentiating conditions and then compared the phosphorylation patterns induced by T_H_17 cell-polarizing conditions. Consistent with the results from the protein array of macrophages, we observed that expression of NF-κB and MAPK signaling intermediates was also suppressed in tIK-Tg mice (Fig. 5c). Furthermore, A20, an inhibitor of the activation of NF-κB^18^, was highly expressed in CD4^+^ T cells from tIK-Tg mice under T_H_17 cell-polarizing conditions, and this was associated with reduced signaling through the NF-κB and p38 pathways (Fig. 5d). In addition, the promoter activities of NF-κB and IL-6 were also significantly lower in tIK-transfected Jurkat T cells than in pcDNA3.1-transfected cells at 18 h after stimulation (Fig. 5e).

### The level of tIK cytokine is higher in the synovial fluid of RA patients

**Figure 6:**
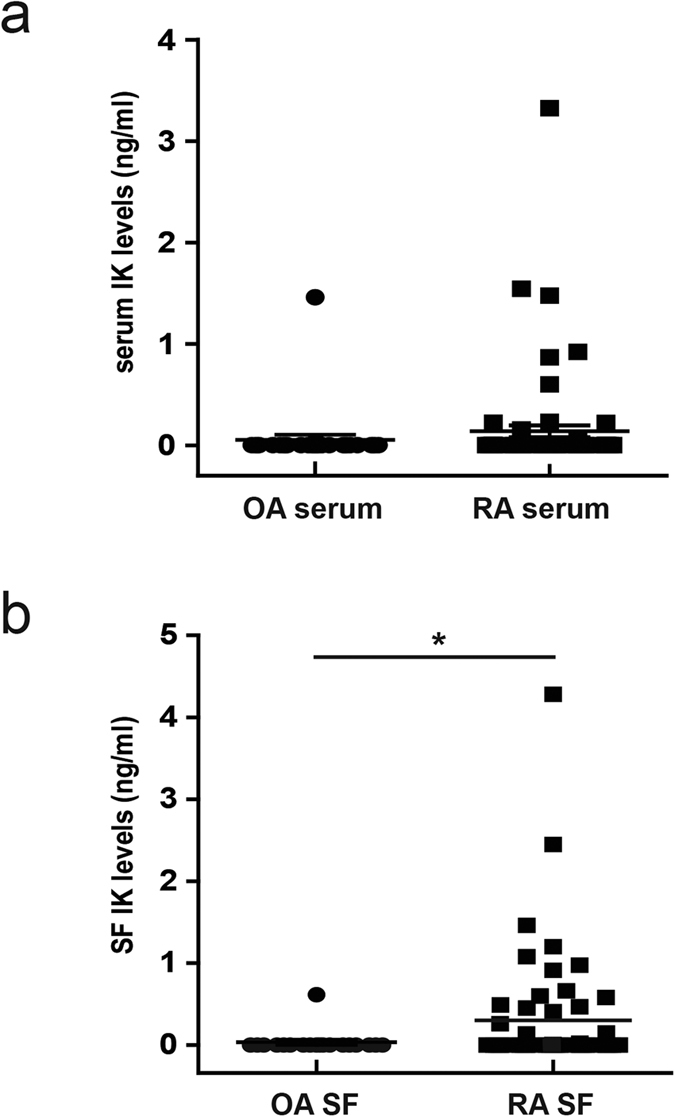
Serum and synovial fluid (SF) levels of IK in patients with rheumatoid arthritis (RA) and subjects with osteoarthritis. (**a**) Serum concentrations of IK were measured in patients with osteoarthritis (OA; n = 29) and RA (n = 70). IK serum level did not differ between RA and OA serum. (**b**) SF concentration of IK was higher in RA (n = 56) than in OA (n = 19). Statistical significance was determined by the Wilcoxon’s test. *P < 0.05.

To demonstrate that tIK cytokine plays a role in human RA, as seen in the mouse disease model, we compared the tIK expression level in serum and synovial fluid (SF) of osteoarthritis (OA) and RA patients. The mean serum IK levels were 0.05 ± 0.27 ng/ml and 0.14 ± 0.48 ng/ml in OA and RA patients, respectively; these were not significantly different (Fig. 6A). IK concentrations in SF were measured in 19 patients with OA and 56 patients with RA. The SF IK levels were significantly higher in patients with RA than in those with OA (0.30 ± 0.72 ng/ml versus 0.04 ± 0.14 ng/ml, *p* < 0.05; Fig. 6B). In patients with RA, IK concentrations in SF were significantly higher than those in serum (*p* < 0.05).

## Discussion

Chronic inflammation associated with RA is critically dependent on an appropriate balance of pathogenic/regulatory immune cells and a break in self-tolerance^2^,^5^,^19^. Although various drugs have been introduced to regulate these immune dysfunctions in RA^3^,^5^, a counterbalancing cytokine that can suppress the symptoms of RA has not been identified. Among the promising immunosuppressive cytokines for treatment of inflammatory diseases, IL-10 is a representative cytokine that is secreted by many cell populations^20^. Numerous investigations have shown that IL-10 plays an important role in the regulation of inflammatory responses as well as in the differentiation and proliferation of immune cells^20^,^21^. For example, IL-10 blocked expression of pro-inflammatory cytokines^22^,^23^. However, IL-10 activates B cells, leading to an increase in autoantibody and serum factor in RA patients^24^. Thus, although IL-10 has therapeutic effects in a mouse model of inflammatory arthritis, it is difficult to apply to human RA.

In this work, we provide substantial evidence for the effectiveness of tIK cytokine as a therapeutic agent for RA. Because IL1Ra is an endogenous inhibitor of IL-1 and is presumed to regulate IL-1 signaling^25^, IL1RaKO mice show excess IL-1 signaling through activation of OX40 on T cells, resulting in the spontaneous development of polyarthritis^14^. In addition, it has been reported that T_H_17 cells are required for the development of arthritis in IL1RaKO mice, while T_H_1 cells have been regarded as influential players that lead to inflamed joint damage by activating macrophages with IFN-γ^14^,^15^,^19^. Thus, IL1RaKO mice are a good model system to analyse the impact of tIK cytokine on pathogenic immune cells during the spontaneous development of arthritis. Interestingly, tIK-IL1RaKO mice showed a reduction in the spontaneous development of inflammatory arthritis, meaning that tIK may have a protective role against the induction of arthritis. Moreover, tIK cytokine suppressed the differentiation and proliferation of both T_H_1 and T_H_17 cells and the functional effects of macrophages by suppressing the NF-κB and MAPK signalling pathways that are activated in immune cells under inflammatory conditions (Fig. 5f). This indicated that tIK cytokine has a homeostatic function in the immune response of inflammatory arthritis.

Finally, we demonstrated higher IK cytokine levels in synovial fluid of RA patients compared with OA patients. This suggested that either the cells that produce IK cytokine are present in the synovial fluid of RA patients but not in that of OA patients, or that IK cytokine is induced in an attempt to regulate inflammation in RA. These data give rise to many questions, including the types of cell that secrete IK cytokine and its effect in the secreting cells. Because the answers to these questions are important for determining the value of IK cytokine as a therapy for RA, further detailed studies are required.

Overall, we demonstrated that tIK cytokine functions as an upstream regulator of inflammatory cascades under stressed conditions. This means that tIK cytokine may have potential for treatment of humans, to be used instead of, or concurrent with, existing disease-modifying anti-rheumatic drugs.

## Materials and Methods

### Mice

tIK transgenic mice were generated as previously described^12^. IL-1 receptor antagonist knockout (IL1RaKO) mice on the BALB/c background were kindly provided by M. L. Cho (Catholic Research Institute of Medical Science, The Catholic University of Korea, Korea). tIK-IL1RaKO mice were produced by crossing tIK-Tg mice and IL1RaKO mice on the BALB/c background. Mice were provided standard food and water *ad libitum.*

### Ethics statement

Mice experiments were carried out in accordance with the relevant guidelines and regulations established by the ethical guidelines and regulations of the Korean Association for Laboratory Animals^26^. All experimental techniques/procedures were approved by the Institutional Animal Care and Use Committee of the Sungsim Campus at the Catholic University of Korea (IACUC Board Regulations #2016-009).

### Cell lines

J774a.1 macrophage cells, Raw 264.7 macrophage cells, and Jurkat T cells (Korean Cell Line Bank) were maintained in RPMI-1640 medium supplemented with 10% FBS (Hyclone) and antibiotics (penicillin and streptomycin; GIBCO).

### Arthritis models

We monitored the spontaneous development of arthritis in IL1RaKO and tIK-IL1RaKO mice over 16 weeks. The incidence of arthritis and the severity score were judged macroscopically and histologically. We scored paws for disease severity on a scale of 0 (no disease) to 4 (maximal swelling) and summed the paw scores to yield individual mouse scores. The joint inflammation scores were evaluated blindly as follows: grade 0, no swelling; grade 1, slight swelling and erythema; grade 2, pronounced swelling; grade 3, joint rigidity; grade 4, maximal swelling. Each limb was graded as a score of 0–4, with a maximum possible score of 16 for each mouse. For histological evaluation, after routine fixation, decalcification, and paraffin embedding of the tissue, we cut joint sections and stained them with hematoxylin and eosin (H&E). We coded all slides and submitted them for evaluation by investigators blinded to the experimental conditions. These investigators judged the extent of synovitis, pannus formation, or bone and/or cartilage destruction as described previously^27^.

### Histopathology of arthritis

The mouse joint tissues were fixed in 10% neutral formalin, decalcified in EDTA bone decalcifier, and embedded in paraffin. Seven micrometer sections were prepared and stained with H&E or safranin O to detect proteoglycans. The sections were deparaffinized using xylene then dehydrated in a graded series of alcohols. The endogenous peroxidase activity was quenched with methanol and 3% H_2_O_2_. Immunohistochemistry for cytokine staining was performed using the Vectastain ABC kit (Vector Laboratories). The tissues were first incubated with anti-TNF-α, anti-IL-1β, anti-IL-17, and goat IgG isotype (for TNF-α) or rabbit IgG isotype (for IL-1β, IL-17) overnight at 4 °C, and then with a biotinylated secondary linking Ab and a streptavidin–peroxidase complex for 1 h. The final color product was developed using 3,3-diaminobenzidine chromogen (DAKO). The sections were counterstained with hematoxylin.

### Microcomputed tomography (micro CT) analysis

The joints were fixed in 10% neutral formalin in preparation for the micro CT imaging, which was performed using an *ex vivo* micro CT scanner (SkyScan 1172; SkyScan). The micro CT scanning parameters were: voltage, 50 kV; current, 201 μA; resolution, 15 μm; and an aluminum filter (0.5 mm). Three-dimensional (3D) images were reconstructed using CT-Vox and CT-Vol software (SkyScan) to produce a visual representation of the results.

### RNA isolation and real-time PCR

Total RNA from the cell pellet, joint, and spleen was isolated using TRIzol reagent (Invitrogen) and first-strand cDNA was obtained using RT&GO (MP Biomedicals), according to the manufacturer’s instructions. We determined the expression of mRNA for *TNFα, IL1β, IL6, IL17, CCR6, NFκBIE, TYK2, IL6R, FCGRIIB, CSF2*, and 18S rRNA (internal control) by real-time PCR using SYBR Green Master Mix (Takara).

### Immunoblot analysis

We fractionated cell lysates by SDS-PAGE, transferred them to polyvinylidenedifluoride membranes and used immunoblotting to analyze them using primary antibodies specific for the following molecules: IκBα (1:1000; Santa Cruz); p-p38 (1:1000; Cell Signaling); p-ERK (1:1000; Cell Signaling); A20 (1:1000; Cell Signaling), and GAPDH (1:3000; Bethyl). The band intensity was analyzed using ImageJ software. The processing (such as changing brightness and contrast) of original blots is appropriate only when it is applied equally across the entire images and is applied equally to controls by Adobe Photoshop.

### ELISA

We measured cytokine concentrations in serum and culture supernatants by ELISA using commercial kits (R&D Systems, eBioscience). We measured the optical density using microplate computer software (Thermo Scientific).

### Flow cytometry

For intracellular cytokine staining, we stimulated splenocytes for 4 h with phorbol 12-myristate 13-acetate (PMA) (50 ng/ml, Sigma) and ionomycin (200 ng/ml, Sigma) in the presence of Golgi Plug (BD Biosciences). We first surface stained the resulting cells with antibodies to mouse CD4 (BD Biosciences) and then stained them with antibodies to mouse IFN-γ (BD Biosciences) and IL-17A (BD Biosciences) or IL-4 (BD Bioscience) using a Cytofix/Cytoperm fixation/permeabilization kit (BD Biosciences) according to the manufacturer’s instructions.

### Microarray

For microarray analysis, we stimulated the splenocytes from WT and tIK-Tg mice with 500 ng/ml LPS O111:B4 (Sigma). The Illumina MouseRef-8 v2 Expression BeadChip was used for transcript profiling; 750 ng of labeled cDNA samples were hybridized to each chip and scanned with an Illumina Bead Array Reader confocal scanner, according to the manufacturer’s instructions (Illumina, Inc., USA). The quality of hybridization and overall chip performance were monitored by visual inspection of both internal quality control checks and the raw scanned data. Raw data were extracted using the software provided by the Illumina GenomeStudio v2009.2 (Gene Expression Module v1.5.4). Array data were filtered by detection p-value < 0.05 (similar to signal-to-noise) in at least 50% of samples. (We applied a filtering criterion for data analysis; a higher signal value was required to obtain a detection p-value < 0.05). The selected gene signal value was logarithm-transformed and normalized by the quantile method. The comparative analysis between test and control samples was performed using fold changes. Microarray data for mRNA were verified by real-time PCR.

### Preparation of bone-marrow-derived macrophages (BMDMs)

For preparation of murine bone-marrow cells, femurs were obtained from 8-week-old WT mice. After euthanasia, the mouse femurs were dissected using scissors, cutting through the tibia below the knee joints and through the pelvic bone close to the hip joint. Muscles connected to the bone were removed using clean gauze, and the femurs were placed into a polypropylene tube containing sterile PBS on ice. The bones were flushed with a syringe filled with cold PBS to extrude the bone-marrow into a 50 ml sterile polypropylene tube. The cell suspension generated was designated fresh bone-marrow cells. Fresh bone-marrow cells were used to generate BMDMs, using L929-cell-conditioned medium (LCCM) as a source of granulocyte/macrophage colony-stimulating factor. The cells were resuspended in 10 ml of bone-marrow differentiation media, which consisted of RPMI-1640 supplemented with 10% FBS (Hyclone), 20% LCCM, 1% antibiotics (GIBCO), and 2 mM L-glutamine. Cells were seeded in Petri dishes (SPL) and incubated at 37 °C in a 5% CO_2_ atmosphere. Three days after seeding the cells, an extra 10 ml of fresh medium was added to each plate and the cells were incubated for an additional 3 days.

### Phosphoprotein array analysis

The Phospho Explorer Antibody Array was performed by Full Moon BioSystems Inc. Cell lysates obtained from BMDM and splenic T cells (treated with LPS; O111:B4, 500 ng/ml or T_H_17 polarizing condition medium, with PBS as a control) were applied to the array, which contains 1318 antibodies recognizing over 30 signaling pathways. Each of the antibodies is present in duplicate and is printed on standard-size coated glass microscope slides. For each antibody, the phosphorylation ratio was calculated using the equation: phosphorylation ratio = (phospho experiment/unphospho experiment)/(phospho control/unphospho control).

### Reporter assay

For macrophages, Raw 264.7 cells were cotransfected with 300 ng of reporter plasmid containing NF-κB luciferase gene. The effector plasmid (450 ng; pcDNA3.1–HA–tIK) or empty pcDNA3.1(+) plasmid and 150 ng of *Renilla* luciferase-expressing plasmid were used. At 48 h after transfection, the cells were stimulated with LPS (O111:B4; 100 ng/ml) for 24 h. For T cells, Jurkat T cells were transfected with GPCR Signaling Pathway Reporter Array kit (Qiagen). The effector plasmid (200 ng; pcDNA3.1–HA–tIK) or empty pcDNA3.1(+) plasmid was used. At 24 h after transfection, the cells were stimulated with anti-CD3 (1 μg/ml) and PMA (10 ng/ml) for 18 h. Cell lysates were assayed for firefly luciferase and then *Renilla* luciferase for normalization, using a dual luciferase assay kit (Promega) according to the manufacturer’s instructions. Luciferase activity was calculated by dividing the firefly luciferase activity by the *Renilla* luciferase activity for each sample.

### T cell differentiation *in vitro*

CD4^+^ naïve T cells from WT and tIK-Tg mice were collected by depleting non-CD4^+^ T cells using the magnetic activated cell sorting (MACS) CD4^+^ T Cell Isolation kit (Miltenyi Biotec). The conditions for inducing the different T helper cell subsets were: 4 ng/ml IL-2 for T_H_0 (neutral conditions); 5 μg/ml anti-IL-4, 5 ng/ml IL-12, and 4 ng/ml IL-2 for T_H_1; 5 μg/ml anti-IFN-γ, 10 ng/ml IL-4, and 4 ng/ml IL-2 for T_H_2; 5 μg/ml anti-IL-4 and 10 ng/ml TGF-β for inducible T regulatory cells; and 5 μg/ml anti-IL-4, 5 μg/ml anti-IFN-γ, 2 ng/ml TGF-β, and 20 μg/ml IL-6 for T_H_17, for 4 days. All cultures were stimulated with 1 μg/ml anti-CD3 and 1 μg/ml anti-CD28.

### Cytokine levels in serum and synovial fluid of OA and RA patients

Serum samples were obtained from 70 consecutive patients with rheumatoid arthritis (RA) and 29 patients with osteoarthritis (OA) who visited the outpatient department, Division of Rheumatology, Bucheon St. Mary’s Hospital, Catholic University of Korea, between March and June 2016. All patients who met the American College of Rheumatology (ACR) criteria for RA^28^ and patients who met the ACR criteria for OA^29^ were included in our study. Synovial fluid (SF) was obtained from 19 patients with OA and 56 patients with RA. Serum and SF samples were stored at −80 °C until analysis. Our study was approved by the institutional review board of Bucheon St. Mary’s Hospital (HC16TISI0070) and was performed in accordance with the principles of the Helsinki II Declaration. All patients gave informed written consent.

### Measurement of IK in serum and SF

IK levels were measured using a commercial sandwich ELISA kit obtained from EIAab (Wuhan, China). To eliminate the influence of rheumatoid factor (RF) on detection of IK levels in ELISA, all sera and SF samples were precleared using a commercial reagent to block heterophilic antibodies (HeteroBlock; Omega Biologicals Inc.).

### Statistical analyses

The experimental values are expressed as mean ± SD. The significance of differences between groups was evaluated using two-tailed unpaired Student’s *t* test or Wilcoxon rank sum test for nonnormally distributed data. GraphPad Prism ver.5 was used for analyses. Differences between groups were considered significant at *P* < 0.05.

## Additional Information

**How to cite this article**: Park, H.-L. *et al*. IK acts as an immunoregulator of inflammatory arthritis by suppressing T_H_17 cell differentiation and macrophage activation. *Sci. Rep.* **7**, 40280; doi: 10.1038/srep40280 (2017).

**Publisher's note:** Springer Nature remains neutral with regard to jurisdictional claims in published maps and institutional affiliations.

## Supplementary Material

Supplementary Data
