# Supplementary material for: IK acts as an immunoregulator of inflammatory arthritis by suppressing TH17 cell differentiation and macrophage activation
Source: Sci Rep. 2017 Jan 10;7:40280. doi: 10.1038/srep40280 (PMC5223115; doi:10.1038/srep40280)
Supplement: Supplementary Data [file srep40280-s1.doc]

# Supplementary information

# IK acts as an immunoregulator of inflammatory arthritis by suppressing T_H_17 cell differentiation and macrophage activation

Hye-Lim Park^1,6^, [Sang-Myeong Lee](http://mail.naver.com/?n=1376878305974&v=f)^2,6^, Jun-Ki Min ^3,6^, Su-Jin Moon^3^, Inki Kim^4^, Kyung-Won Kang^2^, Sooho Park^1^, Seul-Gi Choi^1^, Ha-Na Jung^5^, Dong-Hee Lee^5^, Jae-Hwan Nam^1,*^

^1^Department of Biotechnology, The Catholic University of Korea, Bucheon, 420-743, Korea

^2^Department of Biotechnology, Chonbuk National University, Iksan, 570-752, Korea

^3^Division of Rheumatology, Department of Internal Medicine, College of Medicine, The Catholic University of Korea, Seoul,137-040, Korea

^4^Department of Medicine, College of Medicine, University of Ulsan, Seoul 138-736, Korea.

^5^Biomaterials Research Center, Cellinbio, Suwon, 443-734, Korea

^6^These authors are equally contributed.

^*^Corresponding author : Jae-Hwan Nam

Mailing address : Department of Biotechnology, The Catholic University of Korea, 43-1 Yeokgok 2-dong, Wonmi-gu, Bucheon, Gyeonggi-do, 420-743, Korea

E-mail: [jhnam@catholic.ac.kr](mailto:jhnam@catholic.ac.kr)

Tel: +82-2-2164-4852

**
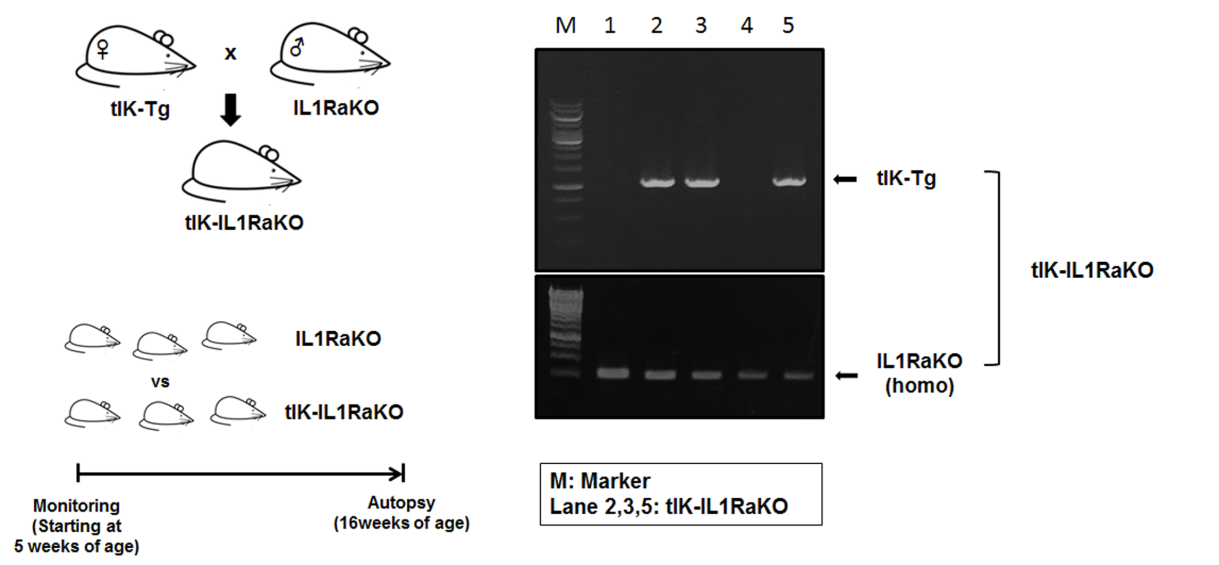
**

**Fig. S1. Screening of tIK-IL1RaKO mice.** Genomic DNA was prepared from transgenic mice by tail lysis and PCR was performed with a tIK-specific primer set and an IL1RaKO-specific primer set. Both tIK product and homozygous IL1RaKO product were detected in tIK-IL1RaKO mice.

**
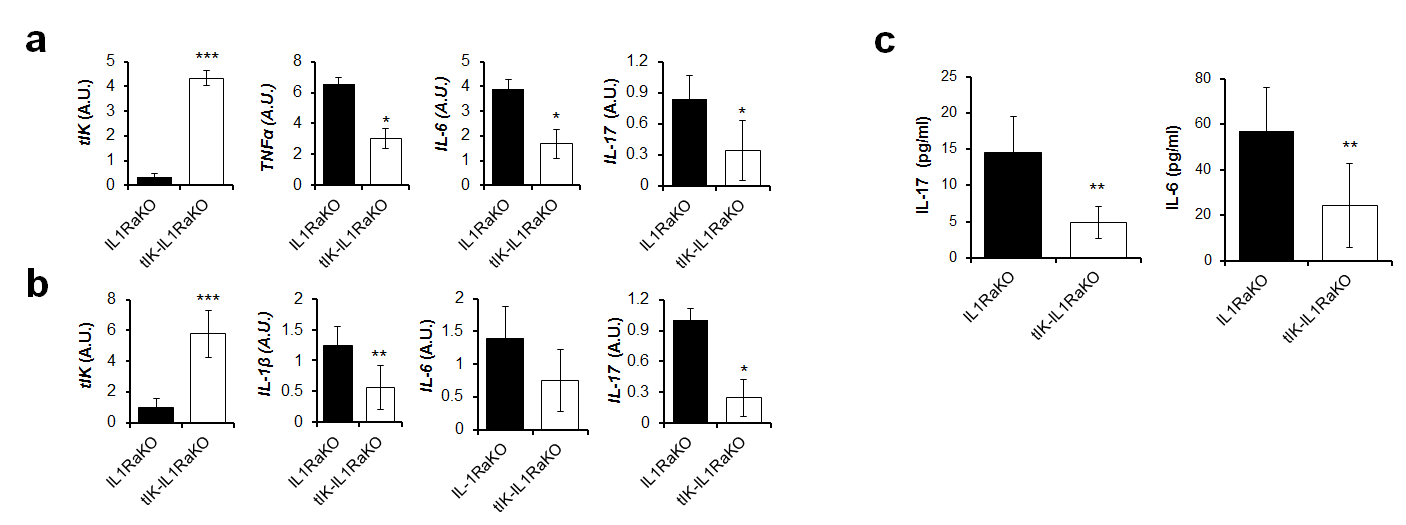
Fig. S2. The levels of inflammatory cytokines in IL1RaKO and tIK-IL1RaKO mice.** (a and b) mRNA expression of inflammatory cytokines in the spleen (a) and joints (b) of IL1RaKO and tIK-IL1RaKO mice (n = 6, mean ± SD). A.U. = arbitrary units. (c) IL-17 and IL-6 levels in the serum of IL1RaKO and tIK-IL1RaKO mice (n = 4–6, mean ± SD).

**
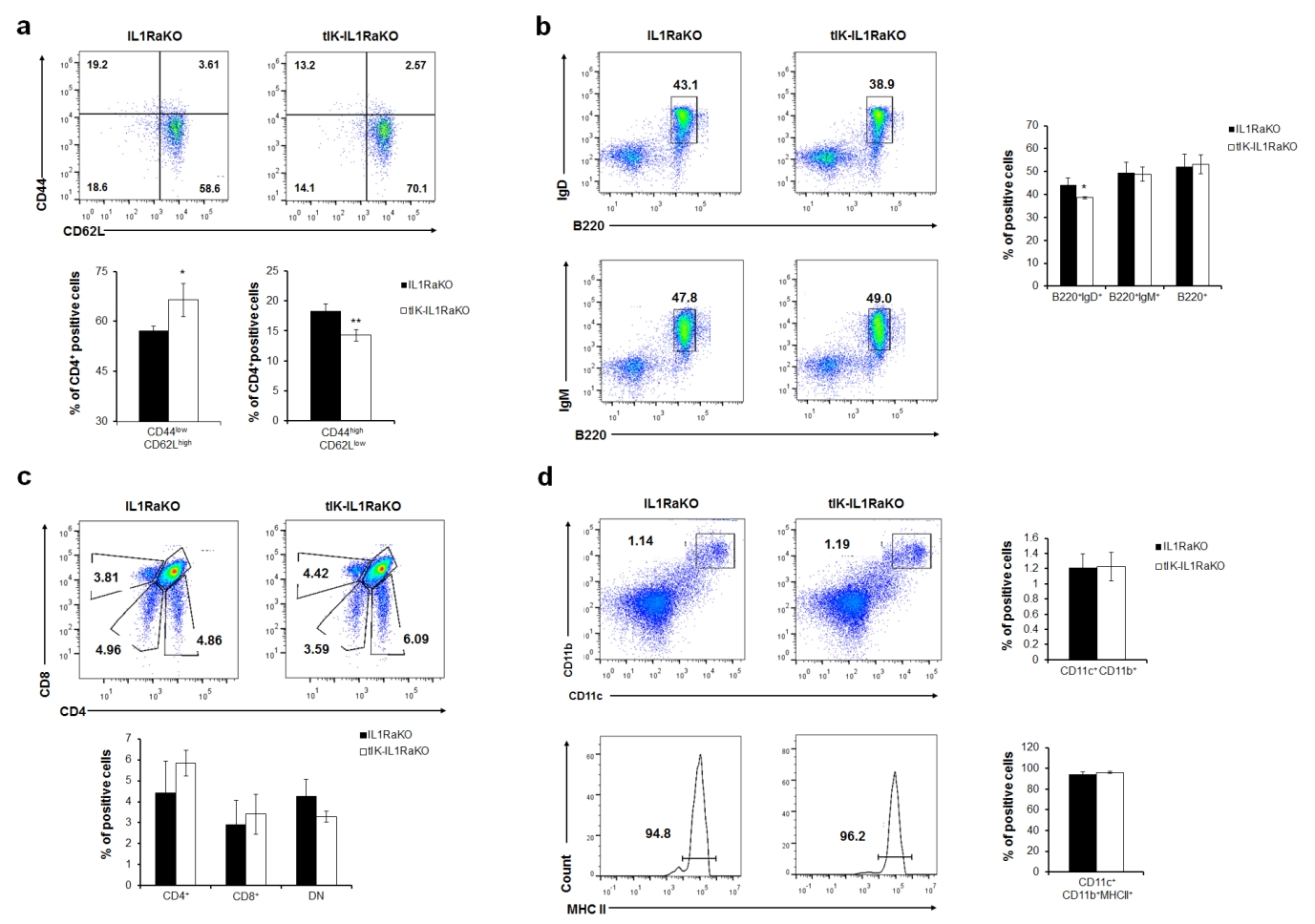
**

**Fig. S3. Immune phenotype of IL1RaKO and tIK-IL1RaKO mice.**

(a) Expression of T-cell-specific surface marker (CD4), effector T cell marker (CD44), and naïve T cell marker (CD62L) in spleens of IL1RaKO and tIK-IL-1RaKO mice. (b) Expression of B-cell-specific surface marker (B220) and B cell development markers (IgM and IgD) in spleens of IL1RaKO and tIK-IL1RaKO mice. (c) Expression of T-cell-specific surface markers (CD4 and CD8) in the thymus of IL1RaKO and tIK-IL-1RaKO mice. (d) Expression of splenic dendritic cell markers (CD11b, CD11c) and MHC class II in IL1RaKO and tIK-IL1RaKO mice. All graphs (a−d) represent the frequencies of gated cells among the total cells (n = 4, mean ± SD). **P* < 0.05, ***P* < 0.01.

**
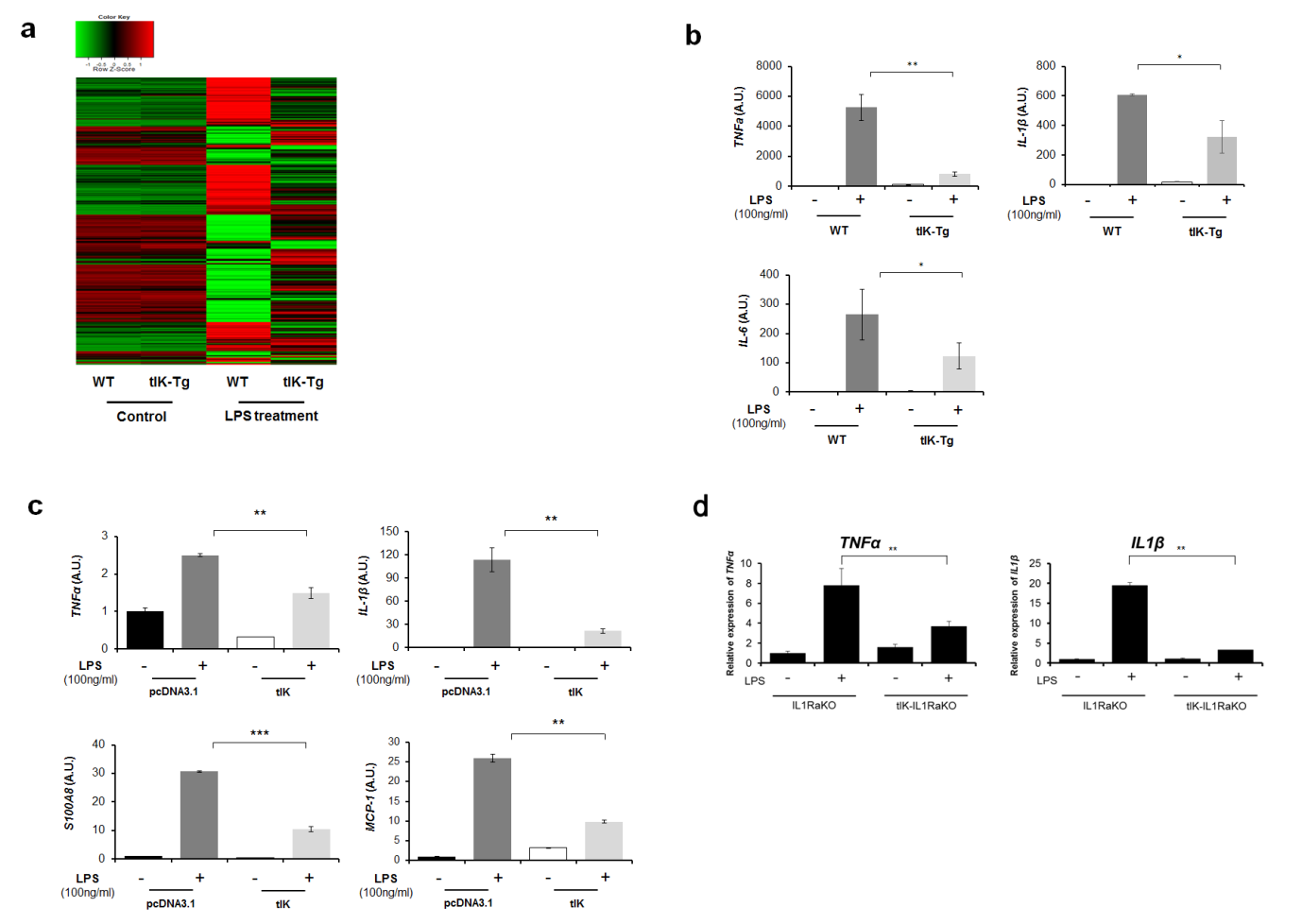
**

**Fig. S4. Effect of tIK cytokine on LPS-induced inflammatory responses.** (a) A heat map showing the fold difference of the expression levels of 3933 genes, showing significant differences between the conditions (fold change ≥ 2), and between WT and tIK-Tg after 500 ng/ml LPS (O111:B4) stimulation. (b) Proinflammatory cytokine mRNA expression in 100 ng/ml LPS-stimulated BMDMs from WT and tIK-Tg mice was analyzed by real-time PCR (n = 3, mean ± SD). (c) Proinflammatory cytokine mRNA expression in 100 ng/ml LPS-stimulated tIK-transfected Raw 264.7 macrophage cells was analyzed by real-time PCR (n = 3, mean ± SD). (d) Proinflammatory cytokine mRNA expression in 100 ng/ml LPS-stimulated splenocytes from of IL1RaKO and tIK-IL1RaKO mice was analyzed by real-time PCR (n = 3, mean ± SD). ‘A.U.’ indicates arbitrary units. **P* < 0.05., ***P* < 0.01., ****P* < 0.001.

**
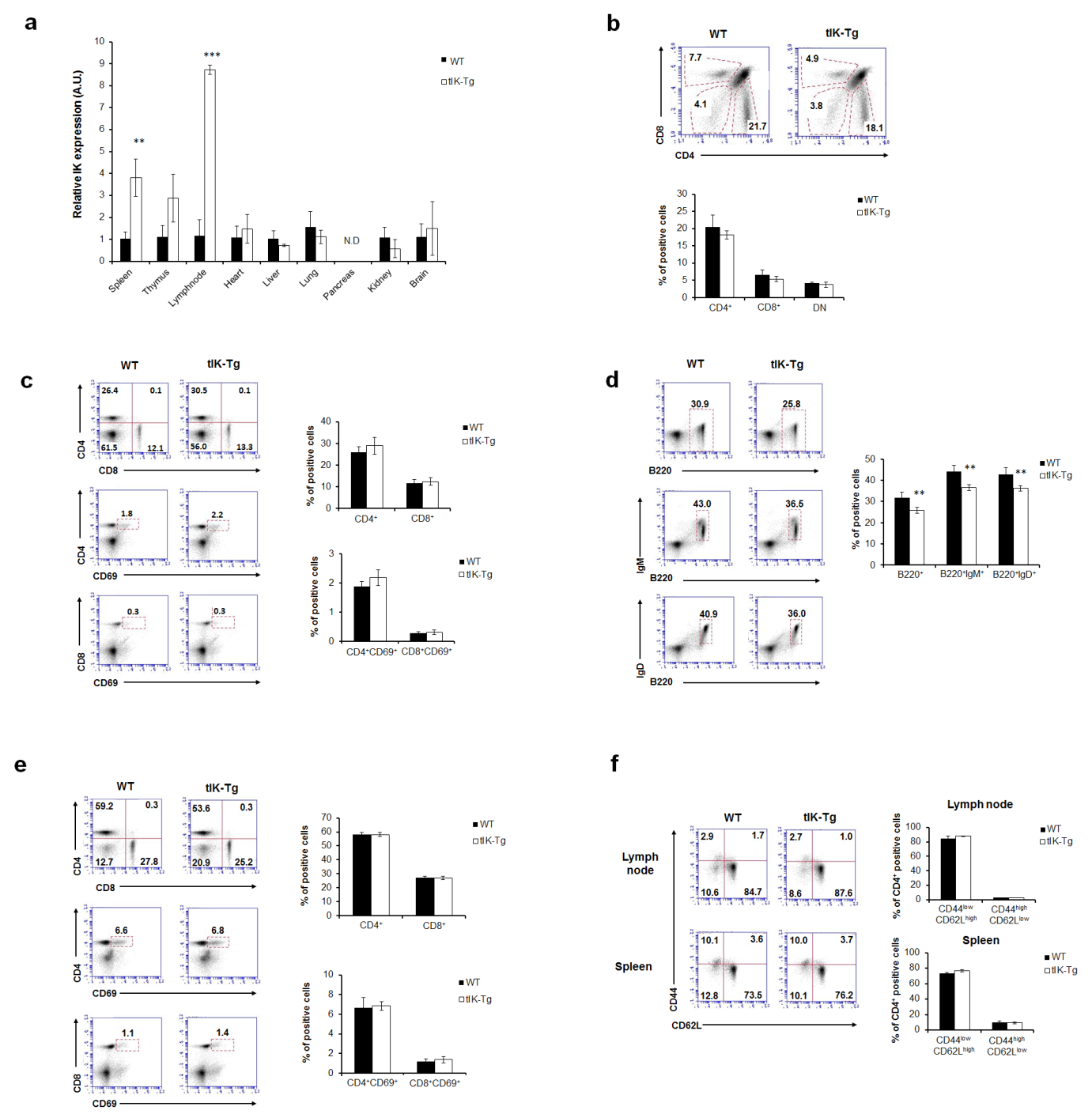
**

**
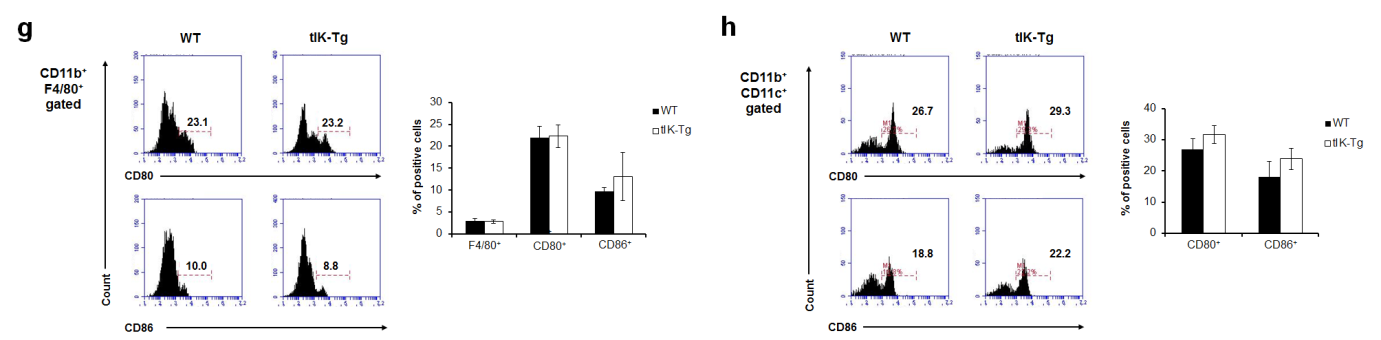
**

**Fig. S5. Immune phenotype of tIK-Tg mice in the steady state.**

(a) Endogenous expression of tIK in tIK-Tg mice. The levels of tIK mRNA were determined by real-time PCR (n = 3, mean ± SD). ‘A.U.’ indicates arbitrary units. (b) Expression of T-cell-specific surface markers (CD4 and CD8) in the thymus of tIK-Tg mice in the steady state. (c) Expression of T-cell-specific surface markers (CD4 and CD8) and early T cell activation marker (CD69) in spleens of tIK-Tg mice in the steady state. (d) Expression of B-cell-specific surface marker (B220) and B cell development markers (IgM and IgD) in spleens of tIK-Tg mice in the steady state. (e) Expression of T-cell-specific surface markers (CD4 and CD8) and early T cell activation marker (CD69) in total lymph nodes of tIK-Tg mice in the steady state. (f) Expression of T-cell-specific surface marker (CD4), effector T cell marker (CD44), and naïve T cell marker (CD62L) in total lymph nodes and spleens of tIK-Tg mice in the steady state. (g and h) Surface expression of splenic macrophage markers (CD11b and F4/80), splenic dendritic cell markers (CD11b, CD11c), and costimulatory factors (CD80 and CD86) in tIK-Tg mice in the steady state. All graphs represent the frequencies of gated cells among the total immune cells (n = 4, mean ± SD). ***P* < 0.01, ****P* < 0.001.

**
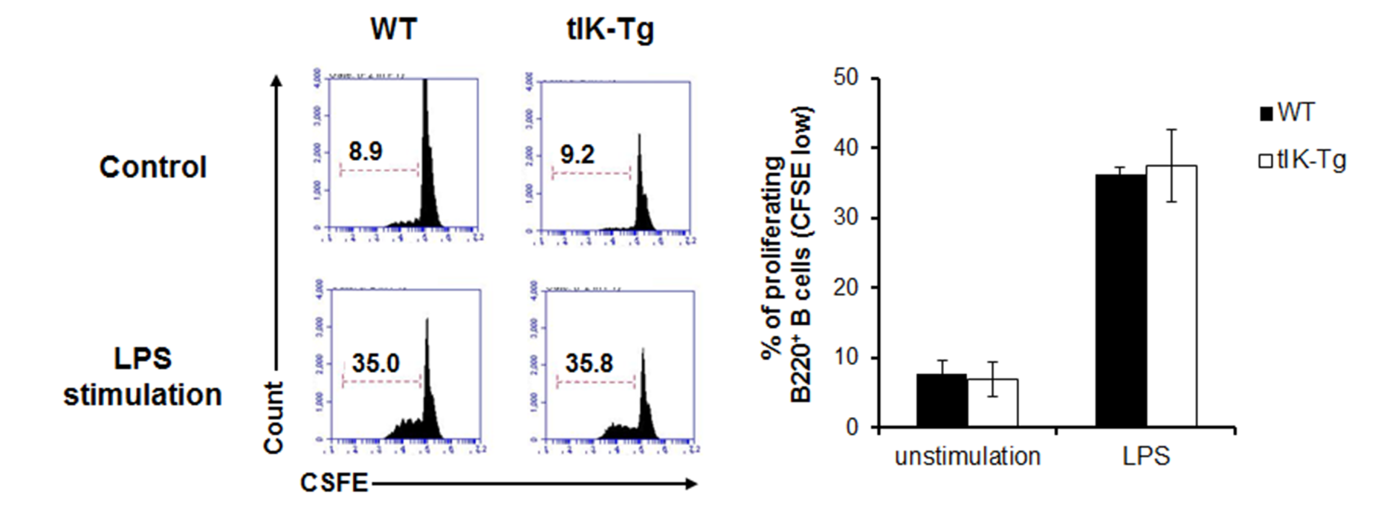
**

**Fig. S6. B cell proliferation in tIK-Tg mice.** Proliferation of B220^+^ splenic B cells from WT and tIK-Tg mice stimulated with LPS (500 ng/ml). Graphs represent the frequencies of gated cells (n = 4, mean ± SD).

**
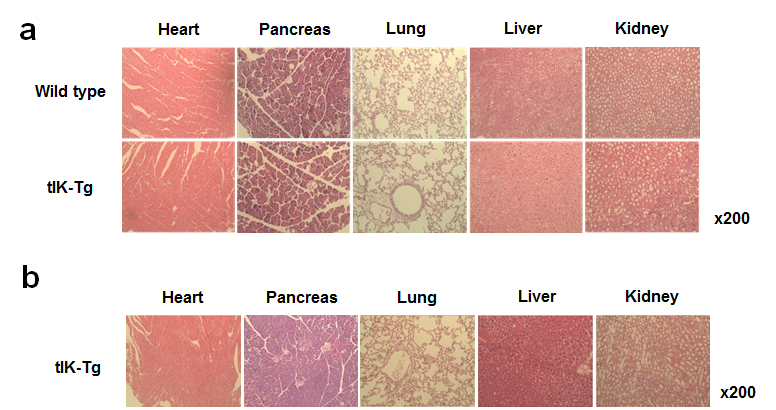
**

**Fig. S7.** **Effect of tIK cytokine on histopathology.** (a) H&E-stained tissue sections of major organs (heart, pancreas, lung, liver, kidney) from WT and tIK-Tg mice at 4 weeks of age (n = 3, original magnification, ×200). (b) H&E-stained tissue section of heart, pancreas, lung, liver, and kidney from tIK-Tg mice at 1 year of age (original magnification, ×200).


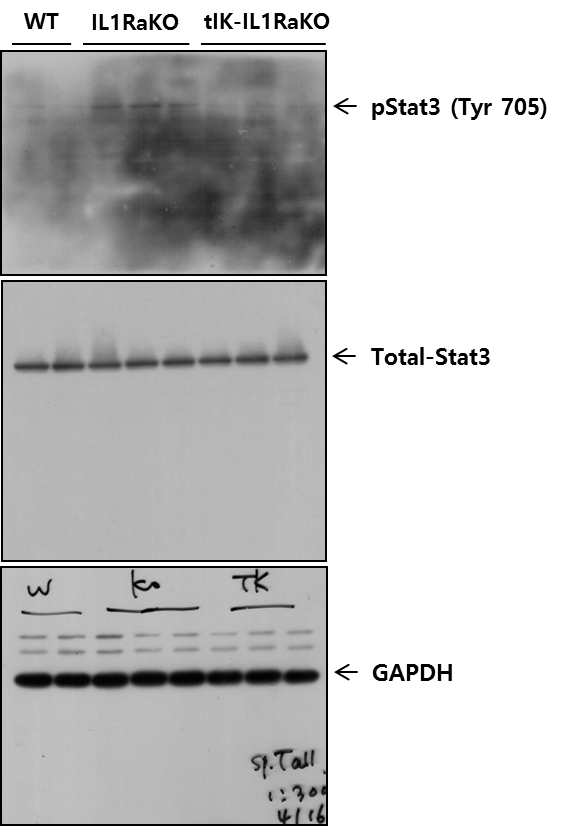


**Fig. S8.** **Full-length blots of Figure 2**


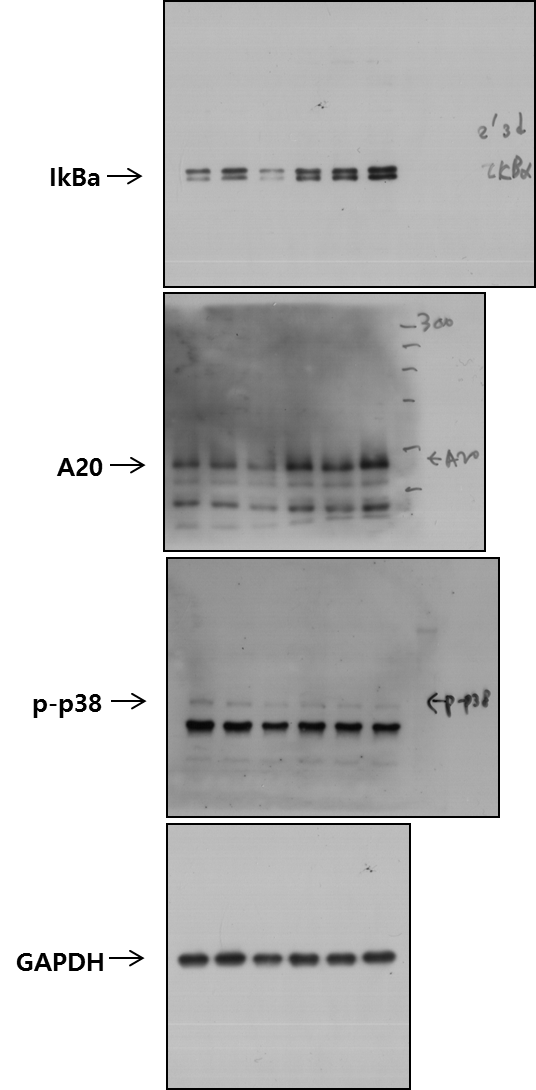


**Fig. S9.** **Full-length blots of Figure 5d**
